# Anti-Melanoma Activities and Phytochemical Compositions of *Sorbus commixta* Fruit Extracts

**DOI:** 10.3390/plants9091076

**Published:** 2020-08-21

**Authors:** Sora Jin, Kyeoung Cheol Kim, Ju-Sung Kim, Keum-Il Jang, Tae Kyung Hyun

**Affiliations:** 1 Department of Industrial Plant Science and Technology, College of Agricultural, Life and Environmental Sciences, Chungbuk National University, Cheongju 28644, Korea; thfkrhehd123@naver.com; 2 College of Agriculture & Life Sciences, SARI, Jeju National University, Jeju 63243, Korea; cheolst0516@gmail.com (K.C.K.); aha2011@jejunu.ac.kr (J.-S.K.); 3 Department of Food Science and Biotechnology, College of Agricultural, Life and Environmental Sciences, Chungbuk National University, Cheongju 28644, Korea

**Keywords:** *Sorbus commixta* fruit, anti-melanoma activity, caspase-3, polyphenolic compounds

## Abstract

*Sorbus commixta* Hedl. (Rosaceae family) has a long history as a medicinal plant in East Asian countries. In this study, we evaluated the effect of *S. commixta* fruit extracts prepared with different ethanol concentrations on anti-melanoma activity, and the extraction yield of phenolic compounds and flavonoids. Using the partitioned fractions from the EtOH extract, we found that the butanol fraction (BF) possessed strong cytotoxic activity against SK-MEL-2 cells (human melanoma cells) but not against HDFa cells (human dermal fibroblast adult cells). Additionally, BF-induced cell death was mediated by the inhibition of the mitogen-activated protein kinase/extracellular regulated kinase (MEK/ERK) signaling pathway, coupled with the upregulation of caspase-3 activity in SK-MEL-2 cells. Furthermore, HPLC analysis of polyphenolic compounds suggested that *S. commixta* fruits contained several active compounds including chlorogenic acid, rutin, protocatechuic acid, and hydroxybenzoic acid, all of which are known to possess anti-cancer activities. Although this study has been carried out by cell-based approach, these results suggest that *S. commixta* fruits contain promising anti-melanoma compounds.

## 1. Introduction

Malignant melanoma, an aggressive and fatal form of skin cancer, is a malignant melanocyte tumor that accounts for approximately 75% of skin cancer-related deaths worldwide [1,2,3]. In recent years, melanoma incidence has continued to increase worldwide, and the onset of malignant melanomas is reportedly affected by various environmental and genetic factors, such as ultraviolet exposure and carcinogenic BRAF mutations [4,5,6]. Despite the wide variety of therapies available to treat melanoma, including surgery, radiation therapy, immunotherapy, and chemotherapy [7,8,9], phytochemicals have been recognized as better anti-cancer therapies to prevent or inhibit carcinogenesis [10]. Camptothecin, vincristine, vinblastine, taxol, topotecan, podophyllotoxin, and irinotecan are good examples of plant-based anticancer molecules [11]. Growing evidence supports that plant-derived natural products are important sources of novel candidates, with pharmacological applicability.

*Sorbus commixta* Hedl. (Rosaceae family) is a well-known medicinal plant used traditionally in East Asian countries including Japan, China, and Korea for the treatment of asthma and other bronchial disorders [12,13]. Additionally, *S. commixta* extracts exhibit a range of biological effects, such as anti-inflammatory [14], antioxidative [15], anti-atherogenic [13,16], vasorelaxant [17], and anti-lipid peroxidation activities [18]. Furthermore, the *S. commixta* fruit also has been used for the treatment of bronchitis and gastrointestinal disorders, as well as for its anti-inflammatory, anti-diabetic, diuretic, and vasorelaxant properties [12,19]. Phytochemical analysis of *S. commixta* fruits has revealed the presence of active ingredients often used in cosmetics, including rutin, isoquercitrin, caffeoylquinic acid, dicaffeoylquinic acid, neosakuranin, chlorogenic acid, neochlorogenic acid, carotenoids, and ascorbic acid [20,21,22], suggesting the potential applicability of *S. commixta* fruits for the production of herbal cosmetics with anti-melanoma activity. Nonetheless, the anti-melanoma properties of *S. commixta* fruits are yet to be comprehensively characterized.

Therefore, our study analyzed the effect of *S. commixta* fruit extracts prepared with different ethanol (EtOH) concentrations on anti-melanoma activity, as well as the role of the mitogen-activated protein kinase/extracellular regulated kinase (MEK/ERK) pathways in the activation of cytotoxicity. Additionally, seven polyphenolic compounds were identified in *S. commixta* fruit extracts via HPLC. Therefore, we expect our study to motivate further interest in the use of *S. commixta* fruits as a natural anti-melanoma source in the cosmetic and pharmaceutical industries.

## 2. Results and Discussion

### 2.1. Effects of S. commixta Fruit Extracts and Solvent Fractions on Human Melanoma SK-MEL-2 Cell

**Figure 1:**
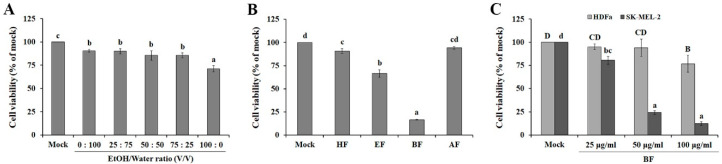
Anti-melanoma activities of *Sorbus commixta* fruit extracts. (**A**) Cytotoxic effects of ethanol concentration on SK-MEL-2 cells. (**B**) Effect of solvent fractions of *Sorbus commixta* fruit ethanol extract on anti-melanoma activity. SK-MEL-2 cells were treated with 100 μg/mL of each extract or solvent fraction. After incubation for 48 h, cell viability was determined via the MTT assay. (**C**) Dose-dependent effect of butanol fraction (BF) on the viability of SK-MEL-2 cells and HDFa human dermal fibroblast adult cells. Dimethyl sulfoxide (DMSO) treated samples served as mock. Values with different letters were found to be significantly different (*p* < 0.05). Values are reported as the mean ± SE. Hexane fraction, HF; ethyl acetate fraction, EF; butanol fraction, BF; aqueous fraction, AF.

Extraction efficiencies are affected by the solvent type and concentration [23]. Particularly, EtOH is considered an excellent solvent and is therefore often used to recover polyphenols from plant matrices, as it is also safe for human consumption [24]. In addition, EtOH is completely miscible with water. Therefore, our study conducted further experiments to define the effect of *S. commixta* fruit extracts prepared with different EtOH concentrations on anti-melanoma activity. As shown in Figure 1A, incubation with 100 μg/mL of EtOH extracts significantly inhibited the proliferation of SK-MEL-2 cells (28.85 ± 3.38%), although ethanol concentration did not have a substantial impact on SK-MEL-2 cell cytotoxicity. Among the bioactive compounds of *S*. *commixta* fruit [22], phenolic and flavonoid compounds have been proposed. Binary-solvent systems have been found to be more effective for the extraction of these compounds from plants compared to mono-solvent systems [25,26]. However, in the case of *S*. *commixta* fruit, EtOH extract contained higher TPC (total phenolic content) levels than water extracts, but solvent concentrations did not significantly affect the extraction of phenolic compound yields. TFC (total flavonoid content) in the extracts increased with increasing ethanol concentrations, and the mono-solvent system examined herein (EtOH) was more effective in extracting phenolic and flavonoid compounds than EtOH/water solvents (Table 1). Additionally, higher concentrations of chlorogenic acid, rutin, protocatechuic acid, and hydroxybenzoic acid in EtOH extracts were observed compared to other extracts, suggesting that the differences in the cytotoxic activity of *S*. *commixta* fruit extracts were due to their concentration and composition of polyphenolic compounds.

Organic solvents such as ethyl acetate (relative polarity 0.228), butanol (relative polarity 0.586), and hexane (relative polarity 0.009) were used to partition the crude extract via the liquid–liquid extraction technique, which is a method to separate compounds, based on their relative solubilities in two different immiscible liquids with different polarities. Therefore, this method is commonly used for the separation of a substance from mixture [27]. It is well known that ethanol (relative polarity 0.654) can dissolve polar compounds, such as sugar, amino acid, glycoside compounds, phenolic compounds with medium polarity, aglycon flavonoids, anthocyanins, terpenoids, flavones, and polyphenols [28], indicating that EtOH extract is a complex mixture of organic compounds. Based on the liquid–liquid extraction technique, highly polar substances, such as organic acids, polysaccharides, free sugars, and proteins stay in the aqueous phase, whereas other relatively less polar compounds, such as terpenoids and polyphenols, are more soluble in organic solvents [29,30]. To enrich the active compounds in *S*. *commixta* fruit EtOH extract, the extracts were partitioned with different solvents, after which the cytotoxicity of each solvent fraction against SK-MEL-2 cells was evaluated. As shown in Figure 1B, the anti-melanoma activities of solvent fractionated EtOH extracts exhibited the following order: BF > EF > HF > AF (83.47%, 33.57%, 9.43%, and 5.56%, respectively). Although several drugs have been used for the treatment of cancer, their cytotoxicity towards normal cells remains a major drawback, which results in secondary malignancy risk [31]. To investigate the effect of BF on the viability of normal cells, HDFa and SK-MEL-2 cells were treated with different concentrations (25 μg/mL, 50 μg/mL, and 100 μg/mL) of BF (Figure 1C). BF exhibited very little cytotoxicity against HDFa cells (5.98%) up to 50 μg/mL, whereas BF showed cytotoxic effects on SK-MEL-2 cells at concentrations of 50 μg/mL, indicating that BF can be a potent source of anticancer agents due to its selective cytotoxicity against melanoma cells. Further experiments were performed at exposure concentrations below (≤50 μg/mL).

Plant-derived polyphenolic compounds (i.e., including flavonoids) have been reported to possess a wide range of pharmacological properties and many polyphenolic compounds have been shown to induce cell cycle arrest and apoptosis in various types of cancer cells [32,33]. Moreover, the anticancer activity of plant extracts has been widely linked to their TPC and TFC. Therefore, our study analyzed TPC and TFC in solvent fractions of *S*. *commixta* fruit ethanol extracts. As shown in Table 2, BF contained the highest amount of phenolic compounds (87.15 ± 4.66 μg GAE/mg of extract), whereas the lowest level of TPC was observed in HF (19.89 ± 2.65 μg GAE/mg of extract). Additionally, total flavonoid levels were measured in the following order: BF (22.25 ± 0.77 μg QE/mg of extract) > EF (13.54 ± 0.43 μg QE/mg of extract) > HF (6.25 ± 1.25 μg QE/mg of extract) > AF (5.96 ± 1.11 μg QE/mg of extract). These results suggested that the inhibitory effect of *S. commixta* fruit solvent fractions on SK-MEL-2 cell proliferation was significantly related to their TFC and TPC. To further identify the active substance that caused SK-MEL-2 cell cytotoxicity, the polyphenolic compounds of the solvent fractions of *S. commixta* fruit were analyzed and quantified via HPLC. We identified and quantified seven polyphenolic compounds, including chlorogenic acid, ferulic acid, gallic acid, hydroxybenzoic acid, protocatechuic acid, rutin, and sinapinic acid (Table 2). BF contained the highest amount of chlorogenic acid (912.72 ± 68.04 μg/g of extract), which is a known antioxidant, anti-inflammatory, and anti-cancer polyphenol compound [34,35]. 

### 2.2. BF Blocks the MEK/ERK Signaling Pathway

**Figure 2:**
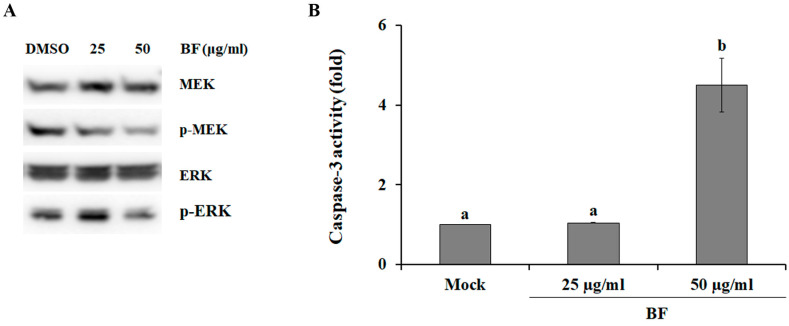
Molecular mechanisms of BuOH fraction (BF)-induced cytotoxicity against SK-MEL-2 cells. (**A**) Effect of the mitogen-activated protein kinase/extracellular regulated kinase (MEK/ERK) signaling pathway on BF-induced cell death. (**B**) Effect of BF on caspase-3 activity in SK-MEL-2 cells. All values are reported as the mean ± SE. DMSO treated samples served as mock. Mean separation within columns was determined via Duncan’s multiple range test at a 0.05% level.

The mitogen-activated protein kinase (MAPK) cascade is an important signaling pathway involved in cellular processes, such as proliferation, and apoptosis. Importantly, dysregulation of MAPK cascades has been linked to several cancers and other diseases [36]. For instance, the MEK/ERK signaling pathway has been shown to play an important role in tumorigenesis and cancer progression [37], suggesting that MEK and ERK are key protein kinases to target for the discovery of anticancer drugs. Therefore, to investigate the involvement of the MEK/ERK signaling pathway in cell death induced by BF, we analyzed the activation level of the MEK/ERK pathway after treatment with BF. As shown in Figure 2A, activation of MEK1/2 and ERK1/2 was inhibited by BF treatment in a dose-dependent manner, indicating that BF reduces the survival of SK-MEL-2 cells by inhibiting the activation of MEK and ERK.

Caspases (aspartate-specific cysteine proteases) are a family of protease enzymes with fate-determining roles involved in many cellular processes, including programmed cell death, differentiation, neuronal remodeling, and inflammation [38]. During apoptosis, caspase-3 (i.e., a major executioner caspase) is cleaved at an aspartate residue to yield a p12 and a p17 subunit to form the active caspase-3 enzyme [39], resulting in the cleavage of key structural proteins, cell cycle proteins, and DNase proteins, such as poly (ADP-ribose) polymerase, gelsolin, ICAD/DFF, and DNA-dependent kinase [40]. When SK-MEL-2 cells were treated with 50 μg/mL BF, caspase-3 activity was strongly increased (Figure 2B), indicating that BF induced cell death via caspase-3 activation. ERK has been found to directly phosphorylate pro-caspase-9 to inhibit caspase-9 processing and caspase-3 activation, resulting in the inactivation of the caspase cascade during apoptosis [41], suggesting that BF increased caspase-3 activity by inhibiting ERK activation in SK-MEL-2 cells. Additionally, chlorogenic acid has been shown to inactivate ERK in hepatocellular carcinoma cells [42]. Furthermore, rutin, which exhibits several promising pharmacological properties including antitumor activity [43], was approximately 22 times more abundant in BF (2.59 ± 0.17 μg/g of extract) than in EF (0.12 ± 0.03 μg/g of extract) and AF (0.12 ± 0.02 μg/g of extract) (Table 2). Taken together, these relationships suggest that rutin and chlorogenic acid might be potential anti-melanoma active compounds in *S. commixta* fruit.

**Figure 3:**
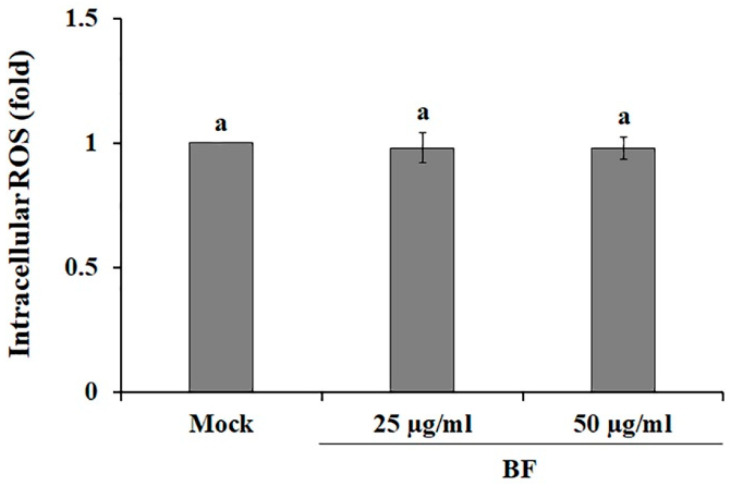
Effects of the BuOH fraction (BF) on reactive oxygen species (ROS) production in SK-MEL-2 cells. ROS level was analyzed using the fluorescent probe DCFH-DA. DMSO treated samples served as mock. Values are reported as the mean ± SE. Values with different letters were found to be significantly different (*p* < 0.05).

In cancer biology, reactive oxygen species (ROS) are known as a double-edged sword, because the imbalance or accumulation of ROS lead to both the survival and death of cancer cells, respectively [44,45]. Particularly, it has been shown that ROS enhances the activation of the Raf/MEK/ERK signaling pathways to promote cancer cell survival, cell proliferation, cell migration, and differentiation [46], whereas ROS accumulation via pro-oxidants under severe oxidative stress conditions leads to apoptosis and cell death [47]. BF-treated SK-MEL-2 cells exhibited similar levels of ROS as compared with mock control (Figure 3), suggesting that inactivation of MEK by BF-treatment (Figure 2A) was not mediated by ROS levels. 

## 3. Materials and Methods

### 3.1. Plant Materials and Sample Preparation

Fresh *S. commixta* fruits were harvested from the research forest at Chungbuk National University, after which the air-dried fruits were ground into a fine powder using a blender. The ground materials were soaked in water, EtOH/water mixtures (25:75 *v*/*v* (EtOH 25%), 50:50 *v*/*v* (EtOH 50%), and 75:25 *v*/*v* (EtOH 75%)), or absolute EtOH for 24 h at room temperature, then sonicated in an ultrasonic bath (1 h × 3 times). The suspension was then filtered and evaporated under reduced pressure, and lyophilized to produce dried powder extract. Additionally, 150 g of the crude EtOH extract was suspended in 1 L of water and sequentially partitioned with hexane (HF, 1 L × 2 times), ethyl acetate (EF, 1 L × 2 times), and n-butanol (BF, 1 L × 2 times). The remaining aqueous extract was used as an aqueous fraction (AF). After filtration, each fraction was evaporated using a vacuum evaporator. 

### 3.2. Cell Viability Assay

SK-MEL-2 human melanoma cells (ATCC^®^ HTB-68™) and HDFa human dermal fibroblast adult cells (ATCC^®^ PCS-201-012™) were purchased from the American Type Culture Collection (ATCC). Cells were cultured in RPMI 1640 medium or DMEM medium supplemented with 10% fetal bovine serum (FBS), 100 U/mL penicillin, and 100 μg/mL streptomycin at 37 °C in a humidified chamber containing 5% CO_2_.

The cytotoxicity of each sample was determined via the MTT assay. Briefly, cells were seeded into 96-well plates at a 5 × 10^3^ cells/well seeding density and incubated at 37 °C in a humidified chamber containing 5% CO_2_ for 24 h. The cells were then treated with the above-described extracts or fractions at a 100 μg/mL concentration. After incubation for 48 h, the medium was replaced with 20 μL of 3-(4,5-dimethylthiazol-2-yl)-2,5-diphenyltetrazolium bromide (MTT) solution (1 mg/mL in PBS) for 4 h. Formazan crystals were dissolved in dimethyl sulfoxide (DMSO), and the absorbance was measured at 520 nm using an iMARK microplate reader (Bio-Rad Laboratories GmbH, Munich, Germany).

### 3.3. Western Blot

After treatment with various BF concentrations for 48 h, SK-MEL-2 cells were analyzed via immunoblotting. The cells were washed with ice-cold PBS and lysed in RIPA buffer (50 mM Tris-HCl pH 7.5, 150 mM NaCl, 1% Triton X-100, 0.1% SDS, 0.5% sodium deoxycholate, 1 mM EDTA, and 10 mM NaF). Protein concentrations were quantified with the Pierce^TM^ BCA Protein Assay Kit (Thermo Fisher Scientific, Waltham, MA, USA). Afterward, 20 μg protein samples were separated via 10% SDS-PAGE and transferred to a PVDF membrane (Millipore, Burlington, MA, USA). After blocking with 5% non-fat dried milk, the membranes were hybridized with MEK1/2, phospho-MEK1/2 (Ser217/221), ERK1/2, and phospho-ERK1/2 (Thr202/Tyr204) (Cell Signalling Technology, Beverly, MA, USA). The signal was detected and visualized via the ECL reagent (SuperSignal™ West Pico PLUS Chemiluminescent Substrate; Thermo Fisher Scientific, Waltham, MA, USA) with an Azure c280 imaging system (Azure Biosystems, Inc., Dublin, CA, USA).

### 3.4. Caspase-3 Activity and Intracellular ROS Measurement

Protein extraction and the analysis of caspase-3 activity were performed using a Caspase-3/CPP32 Fluorometric Assay Kit (BioVision, Milpitas, CA, USA), according to the manufacturer’s instructions. In addition, Intracellular ROS was detected using the DCFH-DA fluorogenic probe as described by Yoo et al. [48]. Caspase-3 activity (400 nm excitation and 505 nm emission) and DCF fluorescence (485 nm excitation and 525 nm emission) were analyzed using a SpectraMax Gemini EM microplate reader (Molecular Devices, San Jose, CA, USA).

### 3.5. Determination of TPC and TFC

TPC and TFC were measured according to the Folin–Ciocalteu and colorimetric methods, respectively, as described by Jin et al. [49] TPC was expressed in milligrams of gallic acid equivalents (mg GAE/g extract) using an equation obtained from a standard gallic acid graph. TFC was determined as milligrams of quercetin equivalents (QE) per gram of extract (mg QE/g extract).

### 3.6. HPLC Analysis

HPLC analysis was performed using a Shimadzu liquid chromatography system (LC-10ADvp) coupled with an ultraviolet-visible detector (SPD-10A; Shimadzu, Kyoto, Japan). The samples were separated with PerkinElmer Brownlee SPP columns (2.7 μm C18 2.1 × 150 mm) at 40 °C at a 0.3 mL/min of flow rate. The mobile phases consisted of water containing 0.1% formic acid (mobile phase A) and acetonitrile containing 0.1% formic acid (mobile phase B). The following gradient elution was performed: 10% B in 0–1.6 min, 20% B in 1.6–11.3 min, 25% B in 11.3–17.8 min, 30% B in 17.8–22.7 min, 60% B in 22.7–25.9, and holding at 80% B in 25.9–28.2 min, the samples were finally reconditioned to the initial conditions. Concentrations were calculated by comparing the sample peak areas with the standard calibration curve.

### 3.7. Statistical Analyses

All experiments were conducted in independent triplicates and significant differences between groups were determined via Duncan’s multiple range test. Values of *p* < 0.05 were considered significant.

## 4. Conclusions

Our study analyzed the effect of different ethanol concentrations on the polyphenolic compound composition and anti-melanoma activity of *S. commixta* fruit extracts. The overall results of the present study suggest that EtOH extract contained a high level of polyphenolic compounds, such as chlorogenic acid, rutin, protocatechuic acid, and hydroxybenzoic acid, all of which are well-known pharmaceutically active compounds, indicating that EtOH extract of *S. commixta* fruits could be a useful source of natural anti-melanoma agents. Additionally, we found that BF induced SK-MEL-2 cell death by increasing caspase-3 activity and inhibiting the MEK/ERK pathway. Taken together, our results provide valuable information for the development of novel anticancer drugs based on *S. commixta* fruit, as well as the optimization of extraction conditions in future studies.

## Figures and Tables

**Table 1:** Effect of ethanol concentration on polyphenolic compound extraction from *Sorbus commixta* fruits.

Polyphenolic Compounds	Water Extract	25% EtOH Extract	50% EtOH Extract	75% EtOH Extract	EtOH Extract
Total phenol content ^1^	50.27 ± 4.2 ^a^	55.71 ± 3.44 ^a^	60.02 ± 9.35 ^a^	64.36 ± 6.35 ^a^	67.98 ± 4.28 ^a^
Total flavonoid content ^2^	2.49 ± 0.25 ^a^	3.53 ± 0.23 ^a^	4.91 ± 0.25 ^b^	6.09 ± 0.21 ^c^	8.65 ± 0.5 ^d^
Chlorogenic acid ^3^	111.81 ± 2.54 ^a^	127.14 ± 1.56 ^b^	150.70 ± 3.40 ^c^	176.72 ± 4.58 ^d^	344.70 ± 7.18 ^e^
Ferulic acid ^3^	0.14 ± 0.02 ^b^	0.09 ± 0.01 ^a^	0.06 ± 0.002 ^a^	0.08 ± 0.01 ^a^	0.13 ± 0.01 ^b^
Hydroxybenzoic acid ^3^	0.13 ± 0.01 ^a^	0.13 ± 0.02 ^a^	0.10 ± 0.01 ^a^	0.10 ± 0.01 ^a^	0.20 ± 0.01 ^b^
Protocatechuic acid ^3^	0.79 ± 0.01 ^a^	0.78 ± 0.09 ^a^	0.92 ± 0.05 ^ab^	1.05 ± 0.03 ^bc^	1.22 ± 0.06 ^c^
Rutin ^3^	ND	ND	0.28 ± 0.06 ^a^	0.48 ± 0.08 ^b^	0.60 ± 0.04 ^b^

^1^ Total phenolic content analyzed as gallic acid equivalent (GAE) μg/mg of extract; values are the average of triplicate experiments. ^2^ Total flavonoid content analyzed as quercetin equivalent (QE) μg/mg of extract; values are the average of triplicate experiments. ^3^ µg/g of extract values are the average of triplicate experiments. Values with different superscripted letters are significantly different (*p* < 0.05). ND = Not detectable.

**Table 2:** Polyphenolic compounds in solvent fractions of *Sorbus commixta* fruit ethanol extract.

Polyphenolic Compounds	Hexane Fraction	Ethyl Acetate Fraction	Butanol Fraction	Aqueous Fraction
Total phenol content ^1^	19.89 ± 2.65 ^a^	72.59 ± 5.34 ^c^	87.15 ± 4.66 ^d^	44.07 ± 2.77 ^b^
Total flavonoid content ^2^	6.25 ± 1.25 ^a^	13.54 ± 0.43 ^b^	22.25 ± 0.77 ^c^	5.96 ± 1.11 ^a^
Chlorogenic acid ^3^	2.20 ± 0.03 ^a^	93.12 ± 14.54 ^b^	912.72 ± 68.04 ^c^	74.20 ± 2.88 ^ab^
Ferulic acid ^3^	0.02 ± 0.004 ^a^	1.24 ± 0.05 ^b^	0.11 ± 0.01 ^a^	0.02 ± 0.001 ^a^
Gallic acid ^3^	ND	0.17 ± 0.02 ^b^	0.04 ± 0.02 ^a^	ND
Hydroxybenzoic acid ^3^	0.36 ± 0.02 ^b^	1.66 ± 0.11 ^c^	0.13 ± 0.01 ^a^	0.04 ± 0.003 ^a^
Protocatechuic acid ^3^	0.05 ± 0.003 ^a^	2.20 ± 0.22 ^b^	2.86 ± 0.01 ^c^	0.26 ± 0.05 ^a^
Rutin ^3^	ND	0.12 ± 0.03 ^a^	2.59 ± 0.17 ^b^	0.12 ± 0.02 ^a^
Sinapinic acid ^3^	ND	0.13 ± 0.01 ^b^	0.07 ± 0.01 ^a^	ND

^1^ Total phenolic content analyzed as gallic acid equivalent (GAE) μg/mg of extract; values are the average of triplicate experiments. ^2^ Total flavonoid content analyzed as quercetin equivalent (QE) μg/mg of extract; values are the average of triplicate experiments. ^3^ µg/g of extract values are the average of triplicate experiments. Values with different superscripted letters are significantly different (*p* < 0.05). ND = Not detectable.
